# Structure and Function of Pancreatic Lipase-Related Protein 2 and Its Relationship With Pathological States

**DOI:** 10.3389/fgene.2021.693538

**Published:** 2021-07-05

**Authors:** Guoying Zhu, Qing Fang, Fengshang Zhu, Dongping Huang, Changqing Yang

**Affiliations:** ^1^Department of Clinical Nutrition, Putuo People’s Hospital, School of Medicine, Tongji University, Shanghai, China; ^2^Department of Pediatrics Gastroenterology, School of Medicine, Washington University in St. Louis, St. Louis, MO, United States; ^3^Department of Gastroenterology, Tongji Hospital, School of Medicine, Tongji University, Shanghai, China

**Keywords:** pancreatic lipase related protein 2, pancreatic lipase, fat digestion, intestinal absorption, polymorphism

## Abstract

Pancreatic lipase is critical for the digestion and absorption of dietary fats. The most abundant lipolytic enzymes secreted by the pancreas are pancreatic triglyceride lipase (PTL or PNLIP) and its family members, pancreatic lipase-related protein 1 (PNLIPRP1or PLRP1) and pancreatic lipase-related protein 2 (PNLIPRP2 or PLRP2). Unlike the family’s other members, PNLIPRP2 plays an elemental role in lipid digestion, especially for newborns. Therefore, if genetic factors cause gene mutation, or other factors lead to non-expression, it may have an effect on fat digestion and absorption, on the susceptibility to pancreas and intestinal pathogens. In this review, we will summarize what is known about the structure and function of PNLIPRP2 and the levels of PNLIPRP2 and associated various pathological states.

## Introduction

Effective digestion and absorption of dietary fats is important, which begins in the stomach by preduodenal lipase with a small amount of dietary triglyceride (Miller and Lowe, 2008). Then, the partially digested emulsion particles empty into the duodenum, where it mixes with the pancreatic lipase secreted from the pancreas. In addition, the common bile duct from the gallbladder merges with the pancreatic duct, supplementing bile salts to the duodenum. The emulsion particles subsequently are hydrolyzed into liquid crystals containing monoglycerides, fatty acids, and cholesterol. Then, the digestion products are transformed by bile salts to the small intestine, taken up by enterocytes, or enter into lymphatic system (Berton et al., 2009; Lindquist and Hernell, 2010). Hence, pancreatic lipase is critical for the digestion and absorption of dietary fats. The most abundant lipolytic enzymes secreted by the pancreas are pancreatic triglyceride lipase (PNLIP or PTL) and its family members, pancreatic lipase-related proteins 1 and 2 (PNLIPRP1/PLRP1 and PNLIPRP2/PLRP2). However, PNLIP is not expressed in neonatal humans and rodents; PNLIPRP1 has no lipase activity (Roussel et al., 1998a; Bakala et al., 2012). Therefore, PNLIPRP2 should have some different properties with PNLIP and plays a pivotal role in lipid digestion, especially for newborns. The purpose of this paper is to review the structure and function of PNLIPRP2 and the relationship between the expression of PNLIPRP2 and various pathological states.

## The Biochemical Properties and Function of PNLIPRP2

**FIGURE 1:**
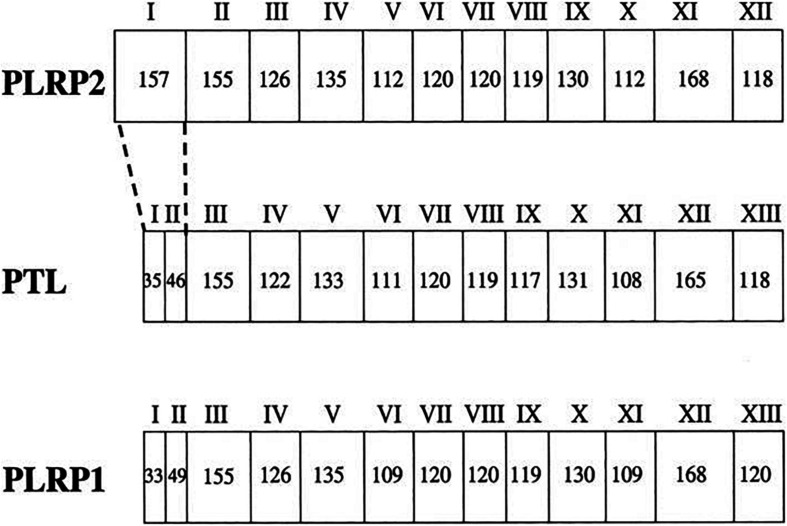
The gene structure for PTL (PNLIP), PLRP1 (PNLIPRP1), and PLRP2 (PNLIPRP2). The exons are numbered with roman numerals. The number of nucleotides in each exon is given in each box. The biggest difference in exons is that PTL (PNLIP) and PLRP1 (PNLIPRP1) both have 13 exons, whereas PLRP2 (PNLIPRP2) only has 12 exons, but it contains a larger exon I (157 bp). PTL (PNLIP), pancreatic triglyceride lipase; PLRP1 (PNLIPRP1), pancreatic lipase-related protein 1; PLRP2 (PNLIPRP2), pancreatic lipase-related protein 2.

PNLIPRP2 (GenBank Accession No. HSA149D17) is a member of the classic triglyceride lipase family. PNLIPRP1 and PNLIPRP2, respectively, have 68 and 65% homologous amino acid sequence with PNLIP (Giller et al., 1992). The biggest difference in exons between PNLIP, PNLIPRP1, and PNLIPRP2 is that both PNLIP and PNLIPRP1 have 13 exons (Figure 1), whereas PNLIPRP2 only has 12 exons, but contains a larger exon I (157 bp) (Lowe, 2000). The larger exon I in PNLIPRP2 is even bigger than the sum of exon I and exon II of PNLIP or PNLIPRP1. The remaining exons are highly conserved (Lowe, 2000). PNLIPRP2 is expressed in various tissues of different species. It was detected in the pancreas of animals including guinea pig, coypu, rabbit, horse, human, and rat; however, it was not expressed in the following species: ox, goat, sheep, dog, and cat (De Caro et al., 2008). The length of human, rat, and mouse PNLIPRP2 mRNAs is 1,500 bp and its protein molecular weight is 53 kDa (Lowe, 2000). PNLIPRP2 lipase has two domains: an N-terminal domain and a C-terminal domain from residues 18 to 353 and 354 to 466, respectively. The N-terminal domain consists of α/β hydrolase fold and the C-terminal has a β-sandwich structure (Ollis et al., 1992). The length of the signal peptide may be 16 or 30 amino acids. It is a 30-amino acid signal peptide in the mouse and rat and a 16-amino acid signal peptide in human and coypu, but less 5′ sequence than the cDNAs isolated from the mouse or rat (Payne et al., 1994; Lowe et al., 1998). Our colleagues’ findings suggest that PNLIPRP2’s expression can be detected before birth, at 15 weeks of gestation in humans and 17 days of gestation in rats and mice (Yang et al., 2000).

**FIGURE 2:**
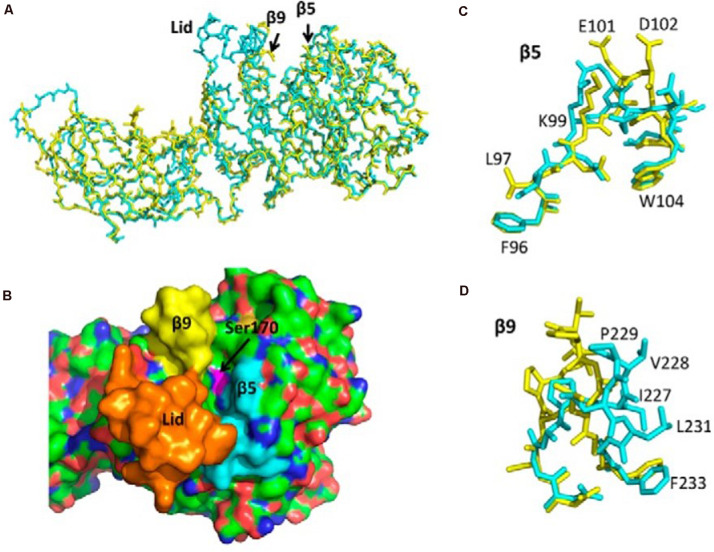
Structure of human PTL (PNLIP) and PLRP2 (PNLIPRP2) and the corresponding lid, β5, and β9 loops. **(A)** Superimposed α-carbon structure of PNLIP (blue) and PNLIPRP2 (yellow). **(B)** Surface structure of PNLIP showing the catalytic site cavity and the location of the lid domain (orange), β5 loop (blue), and β9 loop (yellow). **(C)** Superimposed β5 loops of PNLIP (blue) and PNLIPRP2 (yellow). The labeled amino acids are PNLIPRP2 residues. **(D)** Superimposed β9 loops of PNLIP (blue) and PNLIPRP2 (yellow). The labeled amino acids are PNLIPRP2 residues. PTL (PNLIP), pancreatic triglyceride lipase; PLRP2 (PNLIPRP2), pancreatic lipase-related protein 2.

With the development of X-ray 3D structure technology, scholars can understand the interfacial recognition sites in the molecular structure of these enzymes and the conformational changes in the presence of lipids and amphiphiles (Winkler et al., 1990; van Tilbeurgh et al., 1993). The active sites of many lipases are contained in the N-terminal domain and controlled by a so-called lid formed by a surface loop, β5 loop, and β9 loop. There is a catalytic triad, Ser152-His263-Asp176, at the bottom of this crevice (Berton et al., 2007). This kind of lid makes lipase a special catalytic and interfacial activity at the water/oil interface, but shows low or no activity in a single water and oil phase. In the presence of lipase inhibitors, it undergoes conformational changes, then the solvents in the 3D structure of several lipases can be exposed to the active sites (Egloff et al., 1995; Yang and Lowe, 2000; Eydoux et al., 2008). Nevertheless, the lid structure, β5 loop, and β9 loop act differently among various species. First is the difference between β5 loop and β9 loop, and second is whether the lid is an open conformation or not. Our previous studies show that the structural determinants of human PNLIPRP2 (HPNLIPRP2) lipase activity are the β5 loop and the lid domain, and the β9 loop inversely had smaller effects on activity (Figure 2; Xiao and Lowe, 2015). In contrast, Eydoux et al. (2008) obtained different outcomes by making a crystal structure of HPNLIPRP2 in the absence of amphiphiles and found that the β9 loop is a crucial structural component involved in substrate binding. Dridi et al. (2013) confirmed the role of the β9 loop in the stabilization of the leaving acyl chain in lipolysis reaction on guinea pig PNLIPRP2 (GPNLIPRP2). In addition to the loop structure, another structural determinant of PNLIPRP2 lipase activity is the lid conformation. Eydoux et al. (2008) and our colleagues (Xiao and Lowe, 2015) confirmed that the lid of HPNLIPRP2 adopts an open conformation in solution, contrary to what is observed with the human PNLIP. Therefore, the active site of HPNLIPRP2 might be directly accessible to a substrate. In contrast, the lid of rat PNLIPRP2 (RPNLIPRP2) lipase is in the closed conformation (Mancheño et al., 2004; Valek et al., 2019). Many researchers, including our research group, illustrate the essential role of the lid in determining the substrate specificity and the mechanism of action of lipases (Roussel et al., 1998b; Yang et al., 2000; Berton et al., 2007; Eydoux et al., 2008), and the theory is that closed lid means interfacial activation. Consequently, RPNLIPRP2 lipase displayed interfacial activation at the water/oil interface, while HPNLIPRP2 lipase did not, which was considered a galactolipase. GPNLIPRP2 is the only PNLIPRP2 identified so far with a deletion in the lid domain (Withers-Martinez et al., 1996), but it shows similar kinetic properties with HPNLIPRP2.

Studying the relationship between the structure and function of lipase is of great significance for understanding the role of lipolysis and providing new targets for regulating lipase activity. Although three genes share most of the same structure but differ in their 3D structure and some amino acid sequences, their enzymatic properties are different among them.

## Hydrolyzed Substrate

Substrate specificity is strongly based on the supramolecular organization of the lipid substrates present in oil-in-water emulsions, membranes, micelles, monolayers, or vesicles. PNLIPRP2 had a high activity on all phospholipid–bile salt micelles. They can modify the properties of lipid/water interfaces and promote the enzyme–micelle interaction, thus initiating the effective mass transfer between micelles and enzymes during lipolysis reaction (Mateos-Diaz et al., 2018). PNLIPRP2 has a broader substrate specificity and can hydrolyze triglycerides, phospholipids, and galactolipids. PNLIP has no effect on activity against the phospholipid and galactolipid substrates, except triglycerides (Lowe, 2002; Mancheño et al., 2004), and PNLIPRP1 shows no lipase activity against all known substrates (Roussel et al., 1998).

## Effects of Colipase and Bile Salts on Kinetic Properties

Neonatal and lactating infants express colipase and PNLIPRP2, but not PNLIP. PNLIP is inhibited by normal components of the duodenum, for example, bile acids, phospholipids, or dietary proteins, and colipase can reverse the inhibition of PNLIP (Lowe, 1994; D’Agostino and Lowe, 2004; De Caro et al., 2004; Xiao et al., 2013). The activity of PNLIPRP2 varies greatly among species. HPNLIPRP2 is considered to be a galactolipase, which was inhibited by bile salts when against long-chain triglycerides, with poor activity against diglycerides (Eydoux et al., 2007; Amara et al., 2009; Pang et al., 2011). Horse PNLIPRP2 is the same with HPNLIPRP2 (Jayne et al., 2002b). On the contrary, our research (Xiao et al., 2011) found that HPNLIPRP2 had sufficient activity against long-chain triglycerides and diolein in the presence of bile salt micelles, *in vitro*, and depended on colipase. Under optimal conditions, the activity of mouse PNLIPRP2 (MPNLIPRP2) is about seven-fold greater than HPNLIPRP2 when against long-chain triglycerides and 10-fold higher than HPNLIPRP2 when against short- and medium-chain triglycerides. RPNLIPRP2 and MPNLIPRP2 have activity in the presence of bile salt micelles, and their activity can be increased by colipase (Jennens and Lowe, 1995a, b; D’Agostino and Lowe, 2004). MPNLIPRP2 had full activity in the presence of bovine serum albumin (BSA), whereas BSA completely inhibited MPNLIP except for the presence of colipase (D’Agostino and Lowe, 2004). Why are the effects of colipase and bile salts different among these structurally enzymes? Lowe and Jayne (Jayne et al., 2002a, b; Johnson et al., 2013) suggested that colipase stimulates the activity of PNLIPRP2 by acting on the substrate rather than by anchoring PNLIPRP2 to the substrate interface as the colipase–PNLIP complex does. Therefore, PNLIPRP2 has activity with or without colipase and the degree of activity stimulated by colipase depended on the substrate and PNLIPRP2 species.

## Function of PNLIPRP2

Pancreatic lipase is usually secreted by the pancreas and transferred to the duodenum to participate in the hydrolysis and digestion of fat, cholesterol esters, and fat-soluble vitamins (Carrière et al., 1994). The temporal pattern of PNLIPRP2 mRNA expression confirmed by many experimental data suggests that PNLIPRP2 may play an important role in milk fat digestion in lactating mammals (Li et al., 2007; Andersson et al., 2011). Sucking PNLIPRP2-deficient mice were found to have steatorrhea and fat malabsorption, and the undigested and partially digested triglycerides in feces were significantly increased, accompanied by a significant decrease in weight gain curve (D’Agostino et al., 2002; Huggins et al., 2003; De Caro et al., 2004; Gilham et al., 2007). Intriguingly, as a presumed galactolipase, the main enzyme of HPNLIPRP2 was involved in the digestion of those common vegetable lipids in the gastrointestinal tract, but there is dissimilarity in various species (Bourne et al., 1994; Carrière et al., 1998; Aloulou et al., 2006; Amara et al., 2010). It was detectable in the pancreas of both omnivorous and monogastric herbivorous animals, but not of carnivorous and ruminant herbivorous species, turkey, pigs, and ostrich (De Caro et al., 2008). Galactolipids in the plant kingdom are much more abundant than triacylglycerols, which are ingested by galactolipase-PNLIPRP2. Hence, HPNLIPRP2 likely has some relationships with various races which have diverse component diets.

## Between PNLIPRP2 Levels and Various Pathological States

### PNLIPRP2 Levels and Pancreatitis

More and more lines of evidence show that the expression level of HPNLIPRP2 is related to chronic pancreatitis (CP). It was significantly lower in patients with chronic calcifying pancreatitis (CCP) than in the control group, and the ratio of HPNLIPRP2 to HPNLIP was 23.96% (W/W) and 28.3% (W/W) in CCP patients and controls, respectively (Eydoux et al., 2006). On the contrary, Khatua et al. (2019) found that the expression level of PNLIPRP2 was elevated in fat necrosis and might regulate lipolysis and lipotoxic injury during pancreatitis. The possible explanation is that the secretion of lipase and the occurrence and development of pancreatitis are dynamic processes. Anyway, hPNLIPRP2 is abnormally expressed in subjects with pancreatitis. Intriguingly, more recent studies have shown that genetic variants in pancreatic lipases are associated with an increased risk of CP. One report showed that two brothers with PNLIP deficiency were found to be homozygous for missense mutation in PNLIP and associated with CP (Behar et al., 2014). The PNLIPRP2 W358X (the same with p.W357X and p.W340X) SNP is also of particular interest since it is a common non-sense polymorphism and present in different ethnic groups at a high allele frequency from 0.3 to 0.5. The genetic polymorphism results in a truncated protein, premature truncation of about −24% of the gene product, lacking nearly the entire C-terminal domain of HPNLIPRP2, which is necessary for its stability, efficient secretion, and full activity (Jennens and Lowe, 1995b; Cao and Hegele, 2003). The experience of our research group (Xiao et al., 2013) concluded that the aberrant folding of W358X mutant may cause chronic cellular stress in pancreatic exocrine cells and increase susceptibility to other metabolic stressors. However, Németh et al. (2018) found that the p.W358X truncation variant of HPNLIPRP2 is expressed poorly and has no significant effect on the risk of CP. As a result, it deserved further investigations or more data to elucidate the discrepancy.

### PNLIPRP2 Levels and Other Pathological States

PNLIPRP2 was secreted not only from the pancreas but also from various tissues and cell types under certain conditions, such as cytotoxic T lymphocytes (CTL). It may play an auxiliary role in some types of cytotoxic T-cell-mediated lysis (Alves et al., 2009). Rabbit PNLIPRP2 (also named GP-3) associated with the zymogen granule membranes was detected in enterocytes and Paneth cells (Grusby et al., 1990; Wagner et al., 1994). In the rat hypothalamus, compared with the control group, it was downregulated during fasting (seven-fold) and upregulated (1.8-fold) during conditions of metabolic excess (Rippe et al., 2007). Moreover, it was also regulated by a high-fat (HF) diet at the post-transcriptional level in C57BL/6J mice (Birk et al., 2014). MPNLIRP2 is associated with the hydrolysis of hepatic retinyl esters for the utilization of vitamin A in the mouse liver (De Caro et al., 2004; Reboul et al., 2006) and is responsible for the increased hepatic retinyl ester hydrolases in mice fed vitamin A-deficient diet (Gao et al., 2019). Goat PNLIPRP2 (GoPNLIPRP2) might be regulated by the sexual hormones, because its expression in seminal plasma was significantly increased during the breeding season, parallel to the increase in the plasmatic levels of testosterone (Sias et al., 2005). The low expression of lipases resulted in the delivery of undigested lipid components to the distal ileum, where their intracellular accumulation can lead to the generation of reactive oxygen species (ROS) oxidative stress and the inflammatory characteristics of necrotizing enterocolitis (NEC) (Sodhi et al., 2018). All these data raised the possibility that PNLIPRP2 has other significant functions than just hydrolyzing dietary fats.

## Conclusion

The structural parameters are responsible for the substrate specificity among these structural enzymes, and the degree of activity depends on the substrate and PNLIPRP2 species. These points remain, however, speculations and will deserve further structural studies to determine the conformational state of the PNLIPRP2 lid more precisely and also further investigations to elucidate the molecular mechanisms of PNLIPRP2 processing along with detailed analysis of the digestion products. It might pave the way for exploiting the different expressions and functions of PNLIPRP2 among species, different mutations of PNLIPRP2 among various races, and the relationship between PNLIPRP2 levels and various pathological states and for providing a new drug target to modulate lipase activity.

## Author Contributions

DH and CY conceptualized and supervised the manuscript. GZ, QF, and FZ contributed to the drafting and editing of the manuscript. All authors contributed to the article and approved the submitted version.

## Conflict of Interest

The authors declare that the research was conducted in the absence of any commercial or financial relationships that could be construed as a potential conflict of interest.
